# Correction: *Study protocol for the SARON trial: a multicentre, randomised controlled phase III trial comparing the addition of stereotactic ablative radiotherapy and radical radiotherapy with standard chemotherapy alone for oligometastatic non-small cell lung cancer*

**DOI:** 10.1136/bmjopen-2017-020690corr1

**Published:** 2019-05-09

Conibear J, Chia B, Ngai Y, *et al*. Study protocol for the SARON trial: a multicentre, randomised controlled phase III trial comparing the addition of stereotactic ablative radiotherapy and radical radiotherapy with standard chemotherapy alone for oligometastatic non-small cell lung cancer. *BMJ Open* 2018;8:e020690. doi: 10.1136/bmjopen-2017-020690.

The previous version of this manuscript contains an errors in authors lists that were left out as well as the Contributors statement. It should appear as follows:

John Conibear

Brendan Chia

Yenting Ngai

Andrew Tom Bates

Nicholas Counsell

Rushil Patel

David Eaton

Corinne Faivre-Finn

John Fenwick

Martin Forster

Gerard G Hanna

Susan Harden

Philip Mayles

Syed Moinuddin

David Landau

Kevin Franks

Merina Ahmed

Stephen Harrow

Ruheena Mendes

Paul Hatfield

Yat Man Tsang

Ka Man Mak

Laura Farrelly

Instead of

John Conibear

Brendan Chia

Yenting Ngai

Andrew Tom Bates

Nicholas Counsell

Rushil Patel

David Eaton

Corinne Faivre-Finn

John Fenwick

Martin Forster

Gerard G Hanna

Susan Harden

Philip Mayles

Syed Moinuddin

David Landau

And

DL conceived of the study and is the grant holder. DL, JC, ATB, CF-F, JF, MF, GGH, PM, SM, SH, NC, YN, DE, RP, BC, KF, MA, SH, RM, PH, YMT, KMM and LF initiated, developed and planned the study design. NC also provided statistical expertise in clinical trial design. RP and DE also developed the radiotherapy quality assurance programme for the trial. All the authors contributed to refinement of the study protocol and approved the final manuscript. All contributors have been heavily involved in the design of the trial and have been involved in the drafting and review of the article to be published.

Instead of

DL conceived of the study and is the grant holder. DL, JC, ATB, CF-F, JF, MF, GGH, PM, SM, SH, NC, YN, DE, RP and BC initiated, developed and planned the study design. NC also provided statistical expertise in clinical trial design. RP and DE also developed the radiotherapy quality assurance programme for the trial. All the authors contributed to refinement of the study protocol and approved the final manuscript. All contributors have been heavily involved in the design of the trial and have been involved in the drafting and review of the article to be published.

